# Electrical Properties of ZnO Nanoparticle-Embedded/Polyethylenimine-Functionalized Nitrogen-Doped Graphene Quantum Dot Nanocomposites

**DOI:** 10.1021/acsomega.5c12583

**Published:** 2026-02-25

**Authors:** İlker Yıldız

**Affiliations:** Central Laboratory, 52984 Middle East Technical University, 06800, Ankara, Turkey

## Abstract

In this work, a sustainable solution-based route was employed to synthesize a ZnONPs/PEI N-GQDs nanocomposite by integrating zinc oxide nanoparticles into polyethylenimine-functionalized nitrogen-doped graphene quantum dots. The primary optoelectronic properties of the synthesized material in liquid phase were evaluated by UV–Vis spectroscopy, and Tauc analysis indicated a direct optical band gap of approximately 3.0 eV. Photoluminescence (PL) spectroscopy further revealed a red-shifted visible emission centered at ∼518 nm, attributed to defect- and interface-mediated radiative recombination between ZnONPs and PEI-functionalized N-GQDs. Complementary structural characterization using FTIR, XPS, and TEM confirmed the successful formation of the hybrid nanocomposite, evidencing the coexistence of ZnO nanoparticles and PEI-functionalized N-GQDs. Ultraviolet photoelectron spectroscopy (UPS) was employed to elucidate the interfacial energy-level alignment, yielding a work function of ∼3.17 eV and indicating the formation of a Schottky contact at the metal/nanocomposite interface together with a type-II-like band alignment at the ZnONPs/PEI N-GQDs/n-Si junction. Two diode configurations were fabricated: a conventional Au/n-type Si Schottky diode and a heterojunction diode based on Au/ZnONPs/PEI N-GQDs/n-type Si. Their semilogarithmic current–voltage characteristics were systematically investigated under dark and illuminated conditions at room temperature. Compared with the Au/n-type Si reference, the heterojunction diode exhibited pronounced rectification and a significantly enhanced photoresponse. Illumination induced a substantial increase in reverse current due to photogenerated carriers, resulting in a decrease in the rectification ratio from 3.25 × 10^3^ (dark) to 6.64 × 10^1^ (light) at ±5 V, reflecting a trade-off between rectification and photosensitivity. While the Au/n-type Si diode showed a higher rectification ratio in the dark (2.17 × 10^4^ at ±5 V) and a lower ideality factor (*n* = 5.06), the heterojunction device demonstrated improved illuminated performance with a reduced ideality factor of 3.17 and an increased barrier height of 0.76 eV, underscoring its potential for silicon-based optoelectronic applications.

## Graphical abstract

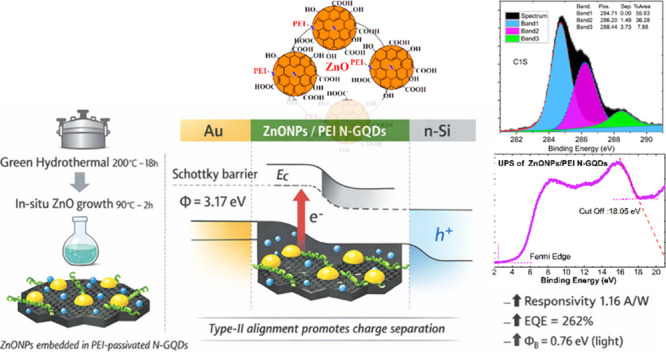

## 1 Introduction

With the continuous advancement of nanotechnology, there is a growing demand for materials that offer tunable, high-performance properties at the atomic scale. Graphene quantum dots (GQDs), as zero-dimensional carbon-based nanomaterials, fulfill this need through their remarkable optical, electronic, and chemical behavior.^1,2^ What distinguishes GQDs from other carbon nanostructures and bulk graphene is their size-dependent, nonzero band gap, which is primarily governed by quantum confinement and edge effects. In particular, nitrogen doping and polymer functionalization further modify the electronic structure, resulting in a small but finite band gap. Structurally, GQDs consist of highly crystalline graphene sheets typically smaller than 100 nm and include both sp^2^ and sp^3^ hybridized carbon atoms along with oxygen-containing functional groups.^3^ These characteristics endow GQDs with excellent photoluminescence (PL), high photostability, biocompatibility, and chemical inertness, making them attractive for a wide range of applications.^4^

To fully exploit these features, various synthesis strategies—ranging from top-down to bottom-up techniques have been developed to precisely control the structure and surface chemistry of GQDs. Among these, heteroatom doping (e.g., nitrogen, boron, sulfur, and phosphorus) has proven particularly effective in tailoring optical and electronic behavior by modifying band structures and charge transport characteristics.^3,5^ In particular, nitrogen doping significantly enhances chemical reactivity and conductivity, enabling broader applicability in areas such as energy storage and environmental remediation.^6,7^

The functional tunability achieved through heteroatom doping has led to significant advances in the design of GQD-based nanostructures for optoelectronic applications. Recent studies have demonstrated that doping can directly influence charge transport behavior and interfacial properties in various device architectures. For instance, gadolinium-doped ZnO quantum dots exhibited enhanced luminescence and a reduced band gap, showing great potential for laser diode applications.^8^ Similarly, incorporating graphene and zinc dopants into organic polymer interfacial layers improved the performance of Schottky barrier diodes.^9,10^ Furthermore, additional gadolinium doping of polyethylenimine-functionalized nitrogen-doped GQDs resulted in a transition from rectifying to ohmic behavior, highlighting the sensitive interplay between dopant chemistry and charge transport mechanisms.^11^ These findings underscore the importance of controlled doping strategies in tailoring the electronic response of GQD-based hybrid systems.

Building on these approaches, the integration of GQDs with zinc oxide (ZnO) nanostructures has emerged as an effective strategy to improve the performance of ultraviolet (UV) photodetectors and solar energy conversion devices.^12^ Au/ZnO quantum dot-based Schottky photodiodes have demonstrated excellent responsivity, contrast ratio, and color selectivity in the UV region.^13^ In addition, incorporating GQDs into ZnO-based architectures enhances photogenerated charge separation and improves transient response and carrier transport dynamics.^14,15^ Complex ZnO morphologies, such as nanoflowers grown on nanorods and decorated with GQDs, exhibit superior UV absorption under weak illumination conditions.^16^ Furthermore, GQD-sensitized GaP/ZnO nanocomposites show high responsivity, detectivity, and external quantum efficiency due to effective band alignment at the GQD–ZnO interface.^17^

Beyond conventional hybrid configurations, recent efforts have focused on direct doping or surface decoration of GQDs with zinc-based species to further expand their optoelectronic versatility. Zn­(II)-doped GQDs exhibit tunable fluorescence in the blue-to-purple range,^18^ while ZnO dimer doping effectively adjusts the electronic properties and band gap of GQDs.^19^ ZnO nanoflowers decorated with GQDs display enhanced photoluminescence across a broad spectral range, making them suitable for flexible optoelectronic platforms.^20^ In addition, codoping strategies, such as nitrogen and sulfur incorporation into GQDs, have improved the performance of quantum dot-sensitized solar cells when paired with ZnO nanorods.^21^ Hierarchical ZnO/GQD composites have also demonstrated enhanced water oxidation kinetics, underlining their catalytic potential.^22^

GQD-functionalized ZnO nanorods have led to substantial improvements in UV photodetector performance, exhibiting enhanced photoresponsivity and detectivity in self-powered photoelectrochemical systems^23^ and Schottky junction-based UV detectors employing GQD-sensitized ZnO/polymer interfaces.^24,25^ These results collectively demonstrate the effectiveness of GQD–ZnO integration strategies in advancing high-performance optoelectronic devices.

In this study, ZnO nanoparticles-doped and polyethylenimine (PEI)-functionalized nitrogen-doped graphene quantum dots (ZnONPs/PEI N-GQDs) nanocomposites were synthesized via a one-step green method and integrated into n-type silicon Schottky barrier diodes. Compared to nonfunctionalized N-GQDs or conventional surface modifiers, PEI introduces distinct interfacial advantages due to its high density of amine groups. Specifically, PEI passivates interfacial defect states, reduces carrier trapping and nonradiative recombination, and induces interfacial dipoles that modify local energy-level alignment at the ZnO/n-Si interface. PEI functionalization introduces amine-rich surface groups that enhance dispersion, improve interfacial electronic coupling, and contribute to the passivation of interfacial trap states compared to nonfunctionalized N-GQDs. Moreover, PEI enhances the dispersion and electronic coupling of N-GQDs within the ZnO nanoparticle matrix, enabling the nanocomposite to function as an active interfacial engineering layer rather than a passive surface coating. To assess their optoelectronic performance, the nanocomposites were deposited onto n-type silicon substrates to form Schottky barrier diodes. The electrical characteristics of the resulting ZnONPs/PEI N-GQDs/n-Si diodes were systematically evaluated under dark conditions, revealing efficient rectifying behavior.

ZnO-based Schottky diodes are known to suffer from interface states, low rectification ratios, and large ideality factors. For example, carbon-dot decoration on ZnO nanorod Schottky diodes improved the rectification ratio from ∼3.13 to ∼17.33 at ±1.2 V, illustrating the strong influence of carbon-based quantum dots on interface engineering and charge transport.^26^ Similarly, incorporating graphene quantum dots or nitrogen-doped GQDs has been reported to enhance electron transport and suppress interface recombination; for instance, PEI-functionalized N-GQDs/p-Si Schottky diodes exhibit rectification ratios as high as ∼2.8 × 10^4^ at ±5 V.^27^ In this context, combining ZnO nanoparticles with PEI-functionalized N-GQDs represents a hybrid junction architecture that remains relatively unexplored and may account for the improved rectifying behavior observed in this study.

Furthermore, green and solution-processed synthesis routes are gaining increasing importance for the development of low-cost and environmentally friendly optoelectronic devices. Functionalized graphene quantum dots have recently been highlighted as versatile interface modifiers, supporting the sustainable strategy adopted in this work.^28^ Although, ZnO/GQD and ZnO/polymer hybrid systems have been widely reported, the present work introduces a distinct interfacial engineering strategy by integrating PEI-functionalized N-GQDs directly within the ZnO nanoparticle matrix rather than employing them as separate interlayers or surface modifiers. This configuration enables simultaneous modulation of interfacial dipoles, defect states, and energy-level alignment at the ZnO/n-Si interface, providing a differentiated contribution beyond conventional ZnO–GQD or ZnO–polymer photodiode architectures. However, despite extensive studies on ZnO/GQD- and ZnO/polymer-based systems, the role of interfacial electronic structure in governing charge transport and rectification behavior in ZnO-based heterojunction photodiodes remains insufficiently understood.

## 2 Materials and Methods

### 2.1 Materials

All chemicals for the synthesis were obtained from commercial sources and used without further purification. The citric acid (CA), Polyethylenimine (PEI) (*M*_w_: 1300, 50 wt % in H_2_O) and ZnCl_2_ were purchased from Sigma-Aldrich. Single side polished (100)-oriented n-type with a resistivity 1–10 Ω·cm and a thickness of 350 μm Czochralski silicon wafer was used for device fabrication.

### 2.2 Synthesis of ZnONPs/PEI N-GQDs Nanocomposites

PEI N-GQDs and ZnONPs/PEI N-GQDs nanocomposites were synthesized using a green method. In green synthesis, the aim is to protect the environment by using nontoxic materials instead of chemicals that are harmful to the environment. In particular, Biological systems such as yeast, fungi, bacteria, and algae, as well as plant extracts, are widely used in green synthesis approaches, where biomolecules present in these systems can mediate reduction and stabilization processes depending on their biochemical composition. A green synthesis approach was employed to prepare the PEI-functionalized nitrogen-doped graphene quantum dots in order to minimize the use of toxic chemicals and reduce environmental impact. In this approach, biological and bioderived components provide functional groups that can facilitate reduction and stabilization processes under mild conditions. Compared to conventional chemical synthesis routes, the green synthetic strategy offers advantages such as simplicity, cost-effectiveness, and improved environmental compatibility, while yielding nanomaterials suitable for optoelectronic applications. In the preparation of metal and metal oxide nanoparticles. In this study, PEI N-GQDs were used as an anion exchanger agent and stabilizer to prepare ZnONPs/PEI N-GQDs nanocomposites.^1,2,27,28^

In the first step, 3.60 g of citric acid (CA) and 7.42 g of polyethylenimine (PEI) were used to synthesize PEI N-GQDs as an anion exchange agent and stabilizer in water in an autoclave at 200 °C for 18 h (Figure 1).^1,2,26,28^

**Figure 1:**
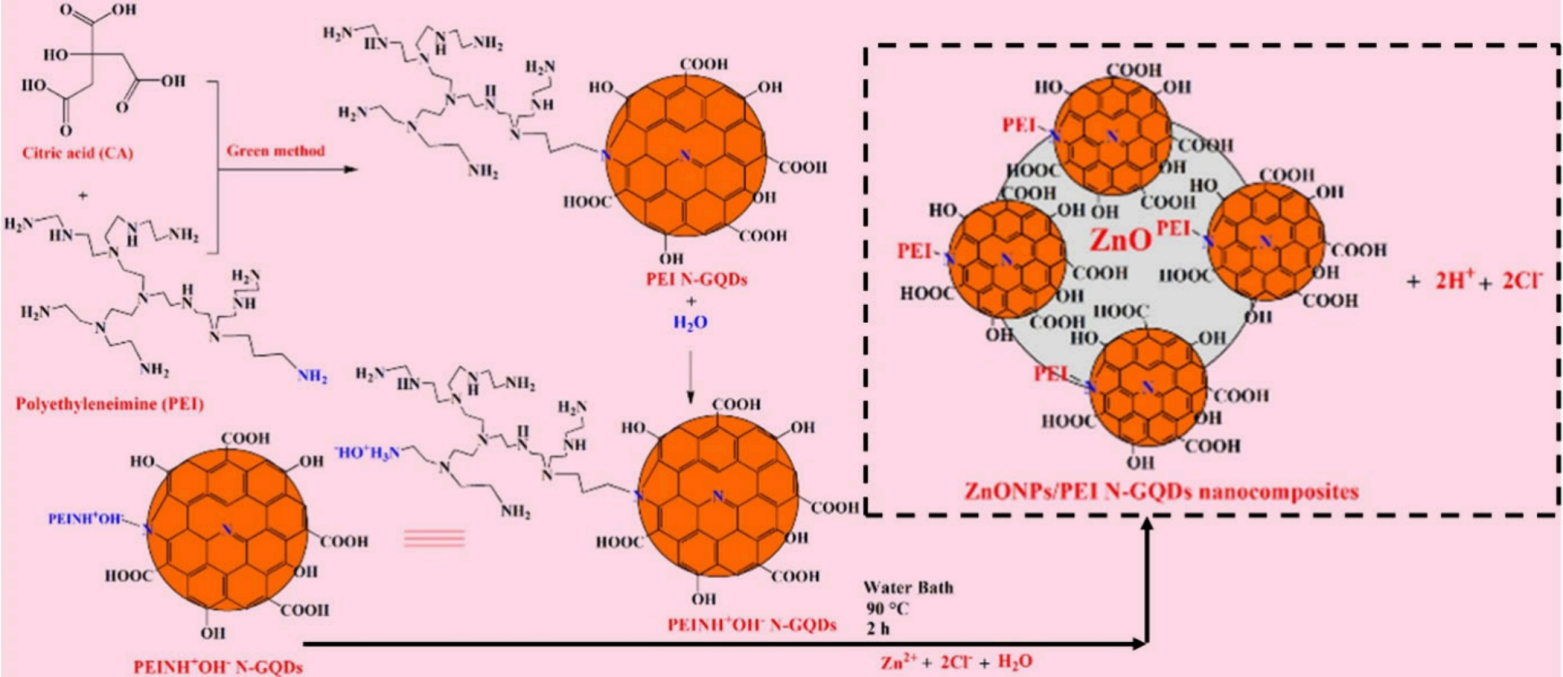
Synthesis of ZnONPs/PEI N-GQDs nanocomposites.

For the synthesis of the ZnONPs/PEI N-GQDs nanocomposite, 2 g PEI N-GQDs solution in 100 mL water and 2 g ZnCl_2_ solution in 50 mL water were added to a 250 mL round-bottom flask. The mixture was heated in a water bath at 90 °C for 2 h with stirring to form ZnONPs/PEI N-GQDs nanocomposites. The mixture was then cooled to room temperature, the suspension products were centrifuged at 12000 rpm for 10 min and the supernatant was collected. ZnONPs/PEI N-GQDs nanocomposites were washed once with deionized distilled water and ethanol. The nanocomposites were stored in a vacuum desiccator for later use.^4,5^

The synthetic route combines a conventional hydrothermal step for the preparation of PEI N-GQDs (200 °C) with a subsequent low-temperature in situ growth process (90 °C) for ZnO nanoparticle incorporation. This two-stage, aqueous, and green method enables uniform dispersion of ZnO nanoparticles on the PEI-functionalized N-GQDs without the use of additional harsh chemicals or organic solvents. As a result, the overall process provides an environmentally friendly, scalable, and energy-efficient method that improves interfacial coupling and enhances the optical and electronic performance of the resulting diode structure.^1,11,26^

### 2.3 Characterization Techniques

To gain insight into the structural and chemical characteristics of the synthesized ZnONPs/PEI N-GQDs nanocomposite, various advanced characterization methods were utilized. These included Ultraviolet–Visible spectroscopy (UV–Vis), Fourier Transform Infrared spectroscopy (FTIR), X-ray Photoelectron Spectroscopy (XPS), Ultraviolet Photoelectron Spectroscopy (UPS), Photoluminescence spectroscopy (PL) and Transmission Electron Microscopy (TEM).

UV–vis spectra were measured using a PG Instruments T+80 UV–visible spectrometer in liquid phase. FTIR spectra were obtained from a PerkinElmer BX II spectrometer in KBr discs and were reported in cm^–1^ units. Elemental compositions of the ZnONPs/PEI N-GQDs nanocomposite materials were analyzed using XPS (PHI 5000 VersaProbeI). The morphological analysis of ZnONPs/PEI N-GQDs nanocomposite was performed using Transmission Electron Microscopy (TEM, Jeol 2100F 200 kV) operated at an accelerating voltage of 200 kV. Prior to imaging, the aqueous nanocomposite suspensions were sonicated in an ultrasonic bath to minimize particle aggregation. A 10 μL aliquot of the well-dispersed suspension was drop-cast onto carbon-coated copper grids (200 mesh) and allowed to dry under ambient conditions. Photoluminescence (PL) measurements were carried out using a Kimmon IK series He–Cd continuous wave laser with a 325 nm excitation wavelength. The excitation beam was focused on the sample at a 45° incidence angle through a 50× objective lens of an optical microscope equipped with a digital camera. The emitted PL signal was resolved using a HORIBA Jobin Yvon iHR550 monochromator equipped with an 1800 grooves/mm VIS holographic grating. Spectral data were collected in extended acquisition mode and recorded with a CCD detector. Ultraviolet Photoelectron Spectroscopy (UPS) measurements were conducted using a He I (21.22 eV) discharge source to determine the work function (Φ).

### 2.4 Device Fabrication

The construction of the ZnONPs/PEI N-GQDs nanocomposite-based device was initiated using a single side polished (100)-oriented n-type silicon wafer, possessing a resistivity 1–10 Ω·cm and a thickness of 350 μm. Prior to nanocomposite deposition, the n-Si substrates were cleaned by sequential rinsing in acetone, alcohol, and deionized water without any additional chemical etching or passivation treatment. To ensure an effective ohmic contact, a 124 nm layer of ultrapure gold (99.999%) was sputtered onto the unpolished rear surface of the wafer. The active nanocomposite layer was then formed by applying the ZnONPs/PEI N-GQDs nanocomposite solution onto the polished front surface via spin coating at 3000 rpm for 30 s, yielding a homogeneous film approximately 30 nm thick (measured by mechanical profilometer from the crater made by hard mask). Following this, top contacts were fabricated by thermally evaporating circular gold electrodes (124 nm thickness) through a metal mask featuring 0.5 mm diameter openings. This process completed the ZnONPs/PEI N-GQDs nanocomposite/n-Si heterojunction device. Around 20 diodes were fabricated on the same substrate, and all exhibited similar rectifying behavior; the device showing the best rectification characteristics was selected for detailed optical measurements. A schematic representation of the overall device architecture is provided in Figure 2.

**Figure 2:**
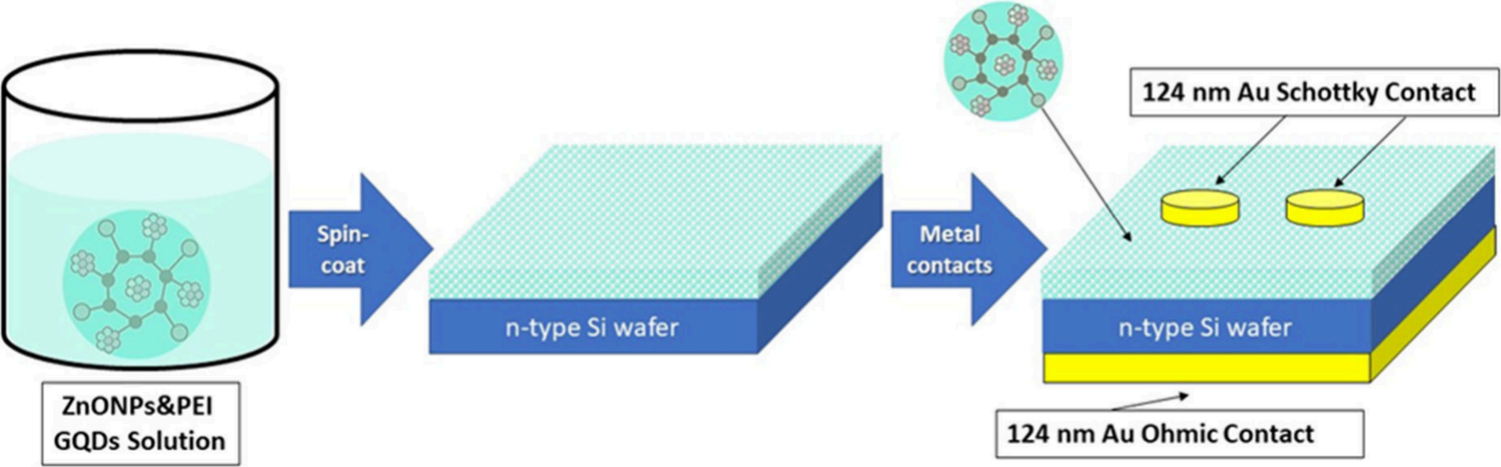
Schematic illustration of the device architecture of the ZnONPs/PEI N-GQDs/n-Si heterojunction photodiode, showing the layered structure and the role of the ZnONPs/PEI N-GQDs nanocomposite as an interfacial layer between the metal contact and the n-Si substrate.

### 2.5 Device Testing Configuration

The ZnONPs/PEI N-GQDs nanocomposite-based device was fabricated by depositing the nanocomposite onto an n-type silicon wafer to evaluate its electrical characteristics. A source-measure unit (Keithley 4200 SCS) was employed to apply bias voltages and record the corresponding current response of the diode. Electrical measurements were carried out under dark conditions at room temperature (300 K), and the semilogarithmic current voltage (*ln*I–V) characteristics were systematically analyzed to assess the rectifying behavior of the heterojunction.

## 3 Results and Discussion

### 3.1 Spectroscopic and Morphological Evaluation of ZnONPs/PEI N-GQDs Using UV–Vis, PL, FT-IR, TEM, XPS, and UPS

The UV–vis absorption spectra of ZnONPs/PEI N-GQDs nanocomposite and PEI N-GQDs were measured in aqueous solution of synthesized primary material and repeated with step by step percentile diluted ones (Figure 3). This dilution aimed as setting the max value of absorption to reach the value 1 for clearance of absorption beyond saturated result. Two slight peaks could observe from the raw data which observed in Figure 3(a). Baseline reduction of a fitted exponential decay background resulted two absorption bands at 258 and 353 nm that could observed in the PEI N-GQDs starting material (Figure 3(b)). The band at 258 nm was attributed to the π–π* transition of C=C and the band at 353 nm to the n-π* transition of C=O and C=N. In the ZnONPs/PEI N-GQDs nanocomposite, a strong absorption at 357 nm and two shoulders at 240 and 276 nm were observed. The band at 258 nm was attributed to the π–π* transition of C=C and the band at 353 nm to the n−π* transition of C=O and C=N. In the ZnONPs/PEI N-GQDs nanocomposite, a strong absorption at 357 nm and two shoulders at 240 and 276 nm were observed, which is consistent with previously reported spectral features of PEI-functionalized N-GQDs.^6^

**Figure 3:**
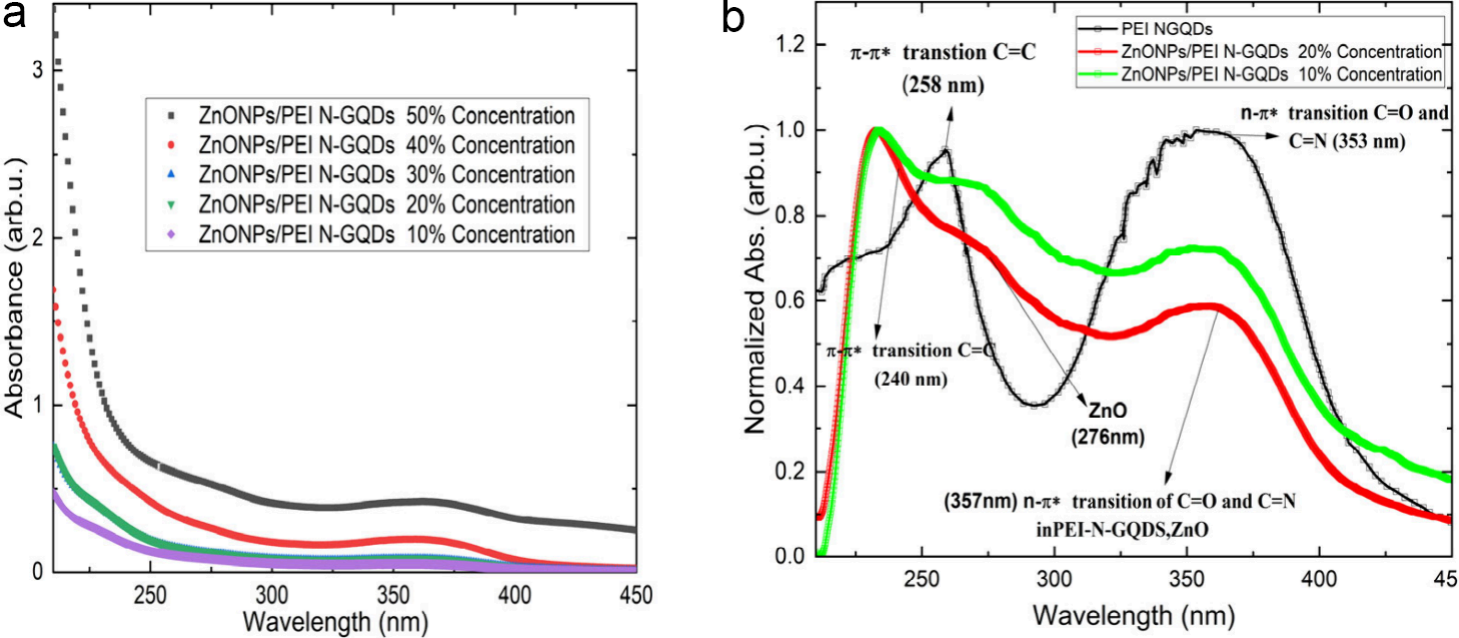
(a) Raw UV–Vis absorption spectra of PEI N-GQDs after dilution to avoid saturation effects. (b) Baseline-corrected absorption spectra showing the intrinsic absorption features.

The strong absorption at 357 nm is attributed to the n−π* electronic transitions of C=O and C=N in PEI N-GQDs, and ZnO. These transitions in ZnO and PEI N-GQDs overlapped. The strong absorption at ∼357 nm is attributed to the n−π* electronic transitions of surface C=O and C=N groups in PEI-functionalized N-GQDs, which typically appear around 357–367 nm in UV–Vis spectra. ZnO nanostructures also show a near-band-edge absorption in the ∼370 nm region.^3^ Therefore, in the ZnONPs/PEI N-GQDs nanocomposite these transitions overlap, giving rise to a merged absorption band around 357 nm.^3,7^

The shoulder at 240 nm belongs to the π–π* transition of C=C in PEI N-GQDs and the shoulder at 276 nm to the ZnO transition. The shoulder observed at about 276 nm appeared as an excitonic absorption peak due to the ZnO nanoparticles being well below the band gap wavelength of 358 nm (3.46 eV).^9^ In the UV–vis spectrum, a ZnO-specific peak is observed between 350 and 370 nm.^9,10^ The optical band gaps of ZnO were measured using UV–vis spectroscopy in water, and 3.1 eV was observed.^9,10^ The optical band gap of the ZnONPs/PEI N-GQDs nanocomposite were determined using the Tauc plot method ­(Figure 6b), and the optical band gap of the ZnONPs/PEI N-GQDs nanocomposite was estimated to be approximately 3.0 eV based on the Tauc plot analysis. These results show that the ZnONPs/PEI N-GQDs nanocomposite is a better conductor than pure ZnO, since the incorporation of PEI-functionalized N-GQDs introduces additional electronic states and interfacial charge-transfer pathways that enhance carrier mobility and suppress recombination, in agreement with previous reports on ZnO/carbon-dot and ZnO/GQD hybrid systems.^11,12^ These results show that the ZnONPs/PEI N-GQDs nanocomposite is a better conductor than pure ZnO, which is consistent with previous studies demonstrating that incorporating carbon-based quantum dots—particularly N-doped graphene quantum dots—into ZnO significantly enhances conductivity by introducing additional electronic states, facilitating charge-transfer pathways, and reducing interfacial recombination.^26,27^ The observed red-shift in the optical band gap is primarily attributed to defect-related states and interfacial hybridization between ZnO nanoparticles and PEI-functionalized N-GQDs. These interactions give rise to localized states near the band edges, effectively reducing the apparent band gap. Quantum confinement effects are expected to play a secondary role in the present system due to the particle size of ZnO nanoparticles.

**Figure 4:**
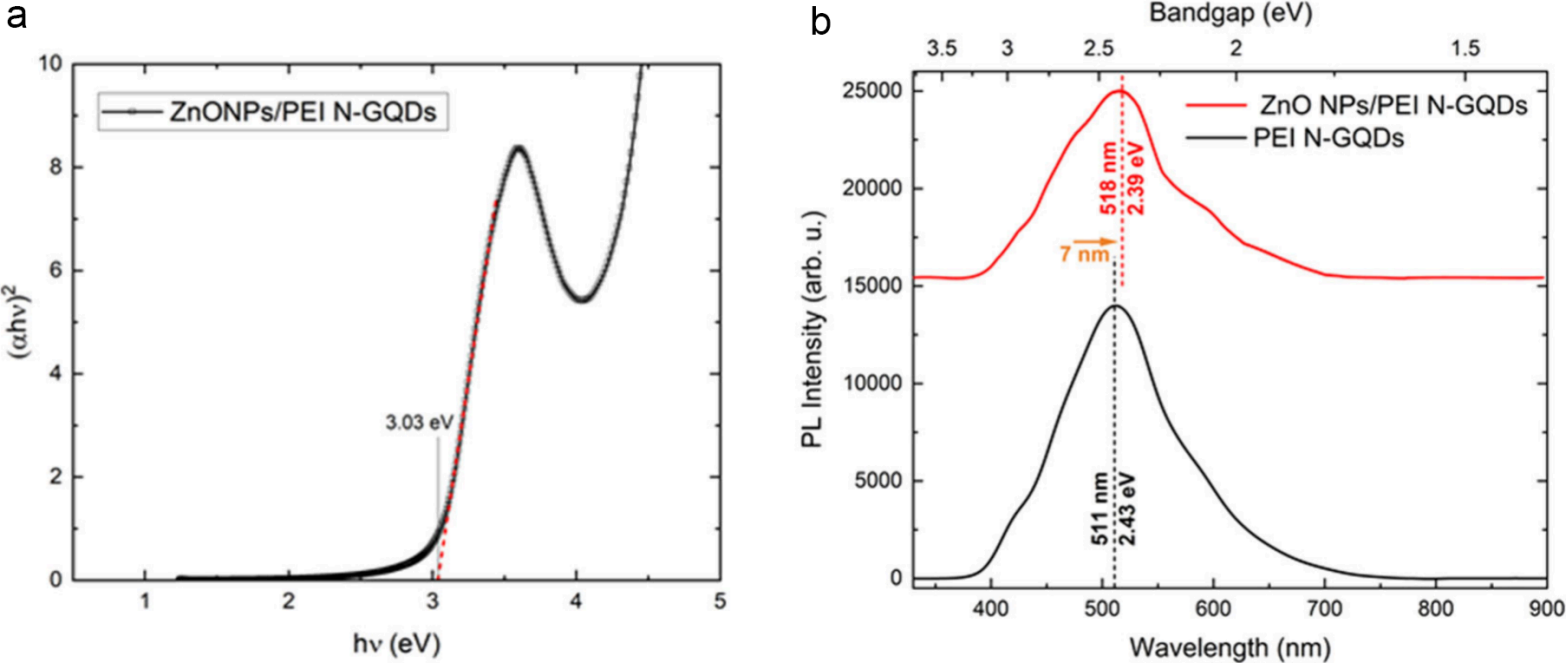
(a) Tauc plot of the ZnONPs/PEI N-GQDs nanocomposite diode, indicating a direct band gap and (b) photoluminescence (PL) spectra showing the optical emission of pristine N-GQDs and the ZnONPs/PEI N-GQDs nanocomposite.

**Figure 5:**
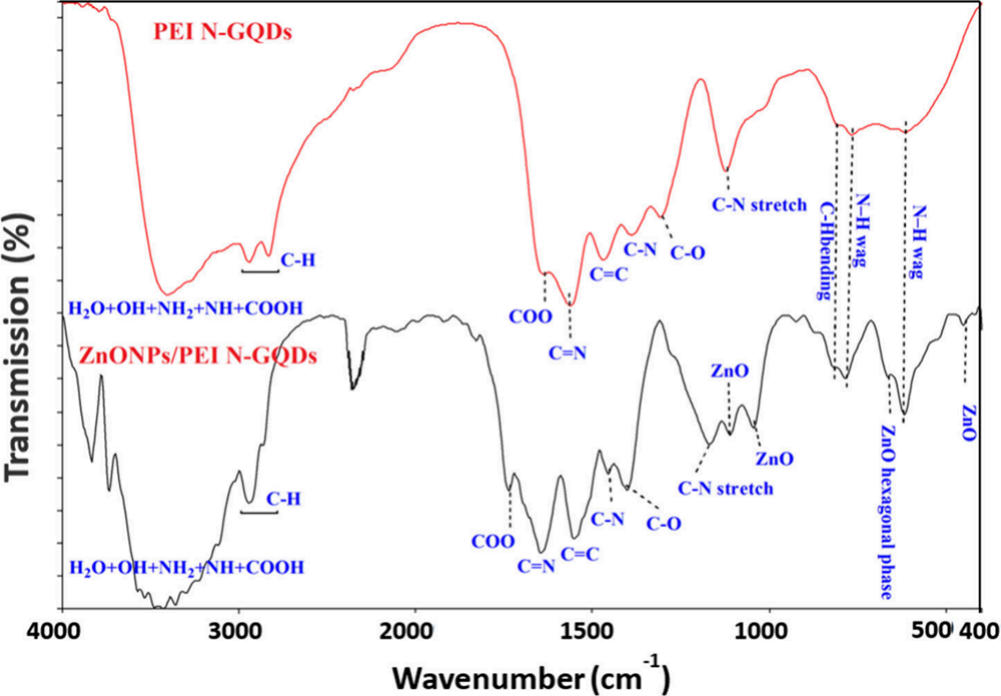
FT-IR spectrum of PEI/N-GQDs and ZnONPs/PEI N-GQDs nanocomposite

**Figure 6:**
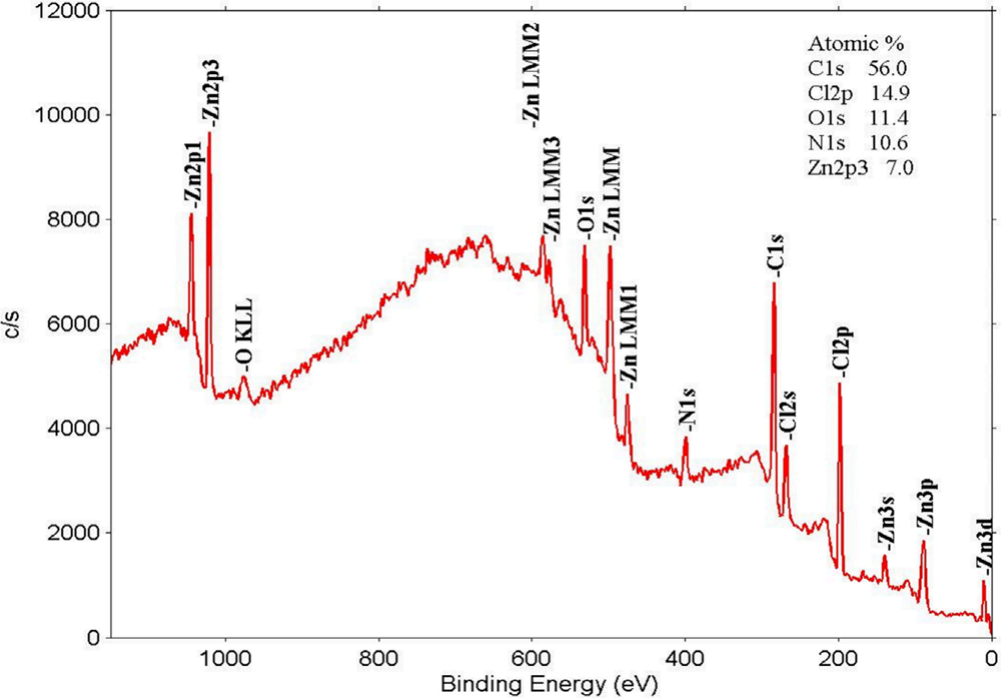
XPS spectrum of the ZnONPs/PEI N-GQDs nanocomposite.

To avoid saturation effects in the UV–Vis measurements, the samples were diluted such that the maximum absorbance remained below 1. The raw absorption spectra (Figure 3a) exhibited a slowly varying background, which was corrected by subtracting a fitted exponential decay baseline. This baseline correction allowed the intrinsic absorption features of the PEI N-GQDs to be more clearly resolved, revealing two absorption bands centered at approximately 258 and 353 nm, as shown in Figure 3b.

When the absorption spectrum of the starting compound PEI N-GQDs material is measured on its own (in free form), π-π* transitions belonging to C=C are observed at 258 nm. The interaction between PEI N-GQDs and zinc chloride results in a new material, the ZnONPs/PEI N-GQDs nanocomposite. Therefore, due to the interaction between the two molecules, ZnONPs and PEI N-GQDs, the π-π* transitions associated with C=C at 258 nm shift to 240 nm in the ZnONPs/PEI N-GQDs nanocomposite. Moreover, the n-π* transitions associated with C=O and C=N observed at 353 nm in PEI N-GQDs shifted to 357 nm in the ZnONPs/PEI N-GQDs nanocomposite. Again, a new shoulder at 276 nm observed in the ZnONPs/PEI N-GQDs nanocomposite belongs to ZnO. This is expected and proves the formation of the new composite material.

The determination of the bandgap is a fundamental preliminary step prior to the development of any potential optoelectronic device. This parameter directly influences subsequent processes such as band bending, charge separation and internal field formation, upcoming at the last of this investigation by UPS evaluation. Hereby, Figure 4(a) presents Tauc plot of the UV–vis spectra for the ZnONPs/PEI N-GQDs nanocomposite, derived from the absorption data proposing a direct electronic transition. The optical band gap of the ZnONPs/PEI N-GQDs nanocomposite was proposed to be approximately 3.03 eV (409 nm), as derived from the Tauc plot assuming a direct transition. Demonstration of bandgap in this case would be proposed by a PL characterization with UV light excitation, that Figure 4 (b) illustrates PL spectrum of the ZnONPs-embedded PEI/N-GQDs nanocomposite. The spectrum displays a broad and intense emission peak centered around 500 nm (∼2.48 eV), indicating strong visible-light emission attributed to radiative recombination through defect or surface states introduced by Zn doping. Figure 4(b) Photoluminescence (PL) spectra of pristine N-GQDs and ZnONPs/PEI N-GQDs nanocomposite. The N-GQDs exhibit a dominant emission at ∼511 nm, while the nanocomposite shows a red-shifted peak at ∼ 518 nm. The spectral shift is attributed to interfacial charge transfer and defect-assisted radiative recombination involving ZnO-related states and PEI-stabilized N-GQD emission centers. Figure 4 (b) To trace the peak lying under the 220–260 nm area I subtract a baseline of exponential decay for measurements with different concentrations, and choose two results to compare with the PEI N-GQDs to understand the changes in absorbance of the subject.

The optical band gap of GQDs is typically reported in the range of 2.8–3.5 eV, depending on their size, surface functional groups, and synthetic route.^13,14^ Compared to pristine ZnO, which exhibits strong absorption in the UV region (∼387 nm), the ZnONPs/PEI-N-GQDs composite shows a redshift in the absorption edge to ∼409 nm. This shift toward the visible region confirms the effectiveness of GQDs and PEI functionalization in modifying the optical properties of ZnO and broadening its spectral response for optoelectronic applications. The optical band gap obtained from the Tauc plot (3.03 eV) represents the intrinsic electronic transition of the ZnO domains, while the PL emission peak at 2.48 eV arises from radiative recombination through defect-related or interfacial states between ZnONPs and PEI N-GQDs. The lower PL energy therefore reflects sub-band gap emission pathways rather than the fundamental absorption edge.

The PL spectra of N-GQDs and ZnONPs/PEI N-GQDs nanocomposites are shown in Figure 4(b). The N-GQDs exhibit a strong emission at ∼ 511 nm, whereas the nanocomposite displays a slightly red-shifted peak at ∼ 518 nm. The red shift and intensity enhancement originate from interfacial electronic interactions and charge transfer between ZnO and the N-GQDs. The conduction-band minimum (CBM) of ZnO lies above the LUMO of N-GQDs, facilitating electron transfer and radiative recombination through ZnO-related defect states such as oxygen vacancies and Zn–N coordination. PEI passivation further stabilizes these emissive centers, suppressing nonradiative decay and enhancing visible-light emission. These results confirm strong interfacial coupling and are consistent with the literature.^1,3^ The optical band gap of the ZnONPs/PEI N-GQDs nanocomposite was estimated to be approximately 3.0 eV from the Tauc plot assuming a direct electronic transition. The photoluminescence spectrum shown in Figure 4(b) provides complementary information on the optical emission characteristics of the ZnONPs-embedded PEI N-GQDs nanocomposite. The enhanced conductivity of the ZnONPs/PEI N-GQDs nanocomposite compared to pristine ZnO is attributed to the introduction of additional electronic states and interfacial charge-transfer pathways arising from PEI-functionalized N-GQDs, which facilitate carrier transport and suppress recombination, in agreement with previous reports on ZnO/carbon-dot and ZnO/GQD hybrid systems.^26,27^

Prior to the FTIR analysis, the optical properties of the materials were examined through photoluminescence (PL) and Tauc plot evaluations. Both measurements exhibited mutually consistent results, indicating the presence of characteristic electronic transitions and confirming the bandgap behavior expected from PEI N-GQDs and their ZnO-based nanocomposite. The agreement between PL emission features and the Tauc-derived optical bandgap supports the successful modification of ZnO with PEI-functionalized N-GQDs and validates the subsequent structural characterization. The 518 nm emission is commonly attributed to oxygen-vacancy-related defect states. While steady-state PL provides qualitative insight into defect-related emission and interfacial interactions, time-resolved PL measurements would be required for a more quantitative assessment of charge-transfer dynamics.

To elucidate the chemical interactions governing the integration of ZnO nanoparticles with PEI-functionalized N-doped graphene quantum dots, FTIR spectroscopy was employed as a complementary structural characterization technique. FTIR analysis is particularly valuable for identifying the vibrational signatures of both organic functional groups and metal–oxygen lattice modes, thereby providing direct evidence for the successful hybridization within the nanocomposite system.

The FTIR spectra (Figure 5) unequivocally demonstrate the effective functionalization of GQDs with PEI, as indicated by the presence of characteristic N–H/O–H stretching vibrations and C–N stretching bands, alongside the Zn–O lattice modes. The simultaneous observation of these organic and inorganic vibrational features substantiates the formation of the ZnONPs/PEI N-GQDs nanocomposite and confirms the coexistence of both components within a unified hybrid network.

FTIR spectra of PEI N-GQDs and ZnONPs/PEI N-GQDs nanocomposite structures were recorded at 4000–400 cm^–1^ in KBr (Figure 5).^15^ The FTIR results reveal the presence of functional groups and Zn–O vibrations in the starting compound PEI N-GQDs and ZnONPs/PEI N-GQDs nanocomposite.^1,5,6,11,26,28,29^ The observation of the vibrations of functional groups in PEI N-GQDs in the ZnONPs/PEI N-GQDs nanocomposite structure and also the observation of Zn–O vibrations indicate the formation of ZnONPs/PEI N-GQDs nanocomposite.^15^ The FTIR spectra (Figure 5) confirm the successful functionalization of GQDs with PEI by the presence of characteristic N–H/O–H and C–N stretching vibrations together with Zn–O peaks, supporting the formation of ZnONPs/PEI N-GQDs nanocomposites. The broad band between 3837 and 3058 cm^–1^ corresponds to the stretching vibrations of -H_2_O, −OH, −NH_2_, −NH and −COOH groups and confirms the presence of amine, hydroxy and carboxyl functional groups in the ZnONPs/PEI N-GQDs nanocomposite structure.^1,5,6,11,26,28,29^ Furthermore, the strong absorption bands at 1737 and 1647 cm^–1^ are attributed to the presence of PEI N-GQDs in the ZnO nanocomposite. Moreover, C=C, C–N and C–O vibrations were observed at 1555, 1457, and 1403 cm^–1^ in ZnONPs/PEI N-GQDs nanocomposite, respectively. In the starting compound PEI N-GQDs, these vibrations were found at lower frequencies. In PEI N-GQDs, COO, C=N, C=C, C=C, C–N, and C–O vibrations were observed at 1655, 1580, 1470, and 1315 cm^–1^, respectively. Aliphatic C–H vibrations were observed at 2921 and 2835 cm^–1^ in PEI N-GQDs, while 2951 and 2866 cm^–1^ were observed in ZnONPs/PEI N-GQDs nanocomposite. Finally, the absorption peak at 1113–1047 and 454 cm^–1^ corresponds to Zn–O stretching vibration, and the absorption at 667 cm^–1^ corresponds to ZnO hexagonal phase.

Given that the functionalization of GQDs with PEI requires further precise verification of elemental composition, chemical states, and interfacial bonding environments, X-ray photoelectron spectroscopy (XPS) was employed as a robust surface-sensitive technique to elucidate the electronic structure and chemical nature of the ZnONPs/PEI N-GQDs nanocomposite. XPS provides quantitative and chemical-state-specific information, making it essential for confirming the successful integration of organic and inorganic components within such hybrid systems.

By XPS analysis the chemical composition, surface chemical states and binding configuration of the ZnONPs/PEI N-GQDs nanocomposite were investigated (Figure 6). The presence of core elements such as Zn, C, N and O explains the formation of ZnONPs/PEI N-GQDs nanocomposite components which must clarify the formation of the proposed form by deeper analysis of each element with its chemical shifts.

In the XPS spectrum of the ZnONPs/PEI N-GQDs nanocomposite, two distinct peaks were observed for Zn 2p at 1045.87 and 1022.79 eV (Figure 7a and 7b), corresponding to Zn 2p1/2 and Zn 2p3/2, respectively.^8^ This indicates the presence of Zn^2+^ in the composite and indicates that Zn (II) in zinc acetate does not change the oxidation step and only the ligand changes in the formation of ZnO nanocomposite. In the C 1s spectrum, three peaks were observed at 288.44, 286.20, and 284.71 eV (Figure 7c). The peaks observed at 288.44 and 286.20 eV are due to side groups in the graphene molecule and correspond to C–N, C–O, C=O, and O=C–O groups, respectively. The peak at 284.71 eV is attributed to the sp^2^-hybridized carbon (C=C) in the aromatic rings of PEI N-GQDs. In the high-resolution N 1s spectrum of ZnONPs/PEI N-doped GQDs nanocomposite (Figure 7d), two peaks at 400.26 (pyrolytic-N) and 402.28 eV (graphitic-N) were found. It can be concluded from the spectrum that pyrrolic-N is the main component. Again, in the high-resolution O 1s spectrum (Figure 7e), the single peak observed at 532.25 eV is attributed to the presence of O=C–O bonded oxygen in the side group in N-GQDs. As seen from the general XPS spectrum, ZnONPs/PEI N-GQDs nanocomposite also showed Zn 3d, Zn 3p3, Zn 3s, ZnLMM1, ZnLMM, ZnLMM2 and O KLL Auger lines at 10.87, 88.41, 140.31, 483.81, 498.78, 586.61, and 975.70 eV, respectively, in agreement with the literature.^30^

**Figure 7:**
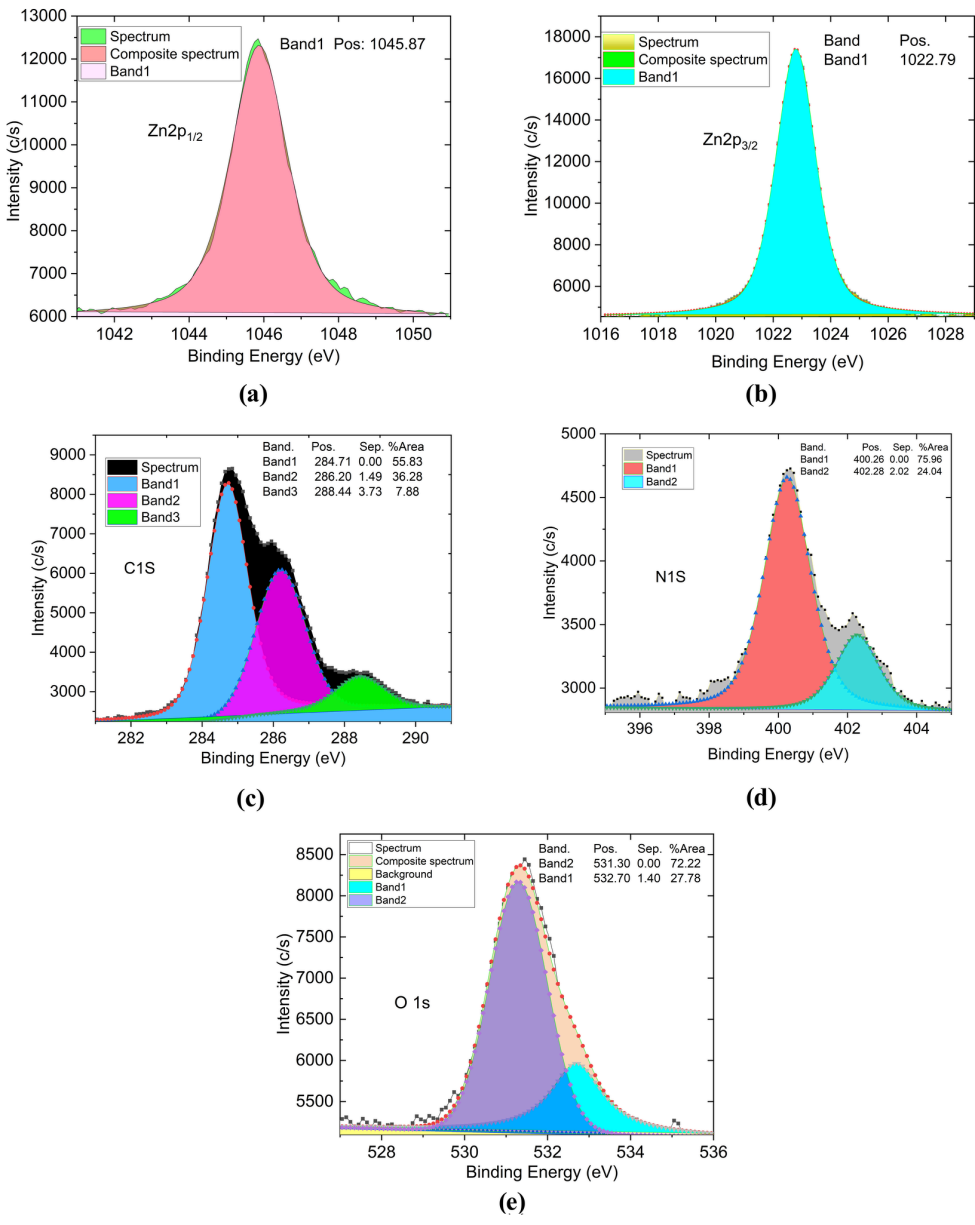
XPS regional spectra of (a) Zn 2p_1/2,_ (b) Zn 2p_3/2,_ (c) C 1s, (d) N 1s, and (e) O 1s peaks for proving the ZnONPs/PEI N-GQDs nanocomposite formation.

For the demonstration of the formation of the ZnONPs/PEI N-GQDs nanocomposite, direct visualization and comprehensive physicochemical characterization are essential. Transmission electron microscopy (TEM) provides critical insight into the morphology, particle size distribution, and spatial arrangement of ZnO nanoparticles within the PEI-functionalized N-GQD matrix, enabling the verification of nanoparticle dispersion and interfacial attachment at the nanoscale. Such imaging is indispensable for confirming the physical integration of the organic and inorganic components, as optical (PL/UV–vis) and spectroscopic (FTIR/XPS) analyses alone cannot reveal morphological details. When combined with FTIR and XPS results that validate the presence of functional groups, chemical states, and bonding environments, TEM observations offer a holistic confirmation of the successful formation of the ZnONPs/PEI N-GQDs hybrid structure.

Atomic level investigation presented by Figure 8 showing the high-resolution transmission electron microscopy (HR-TEM) image of the ZnONPs/PEI N-GQDs nanocomposite. The red circles highlight regions containing lattice fringes corresponding to crystalline ZnO nanoparticles distributed within the nanocomposite matrix while the sizes about 2–5 nm. The inset (upper left) shows the fast Fourier transform (FFT) pattern, while the inset (lower left) displays the selected area electron diffraction (SAED) pattern, both indicating the crystalline nature of the embedded nanoparticles. The contribution of PEI N-GQDs is not clearly resolved in the diffraction patterns due to their semicrystalline/amorphous nature and the dominance of the ZnO diffraction intensity. The surrounding matrix exhibited low-contrast regions without clear lattice fringes, suggesting that the nitrogen-doped graphene quantum dots (PEI N-GQDs) were present in a semicrystalline or amorphous-like carbon phase enclosing the crystalline ZnO nanoparticles. “The measured lattice spacings of ∼0.24–0.26 nm and ∼0.33–0.34 nm correspond to the (101)/(100) planes of hexagonal wurtzite ZnO and the (002)/(100) planes of graphitic N-GQDs, respectively, which are consistent with previously reported studies confirming hybrid ZnO–GQD nanoscale heterointerfaces.^13,17^

**Figure 8:**
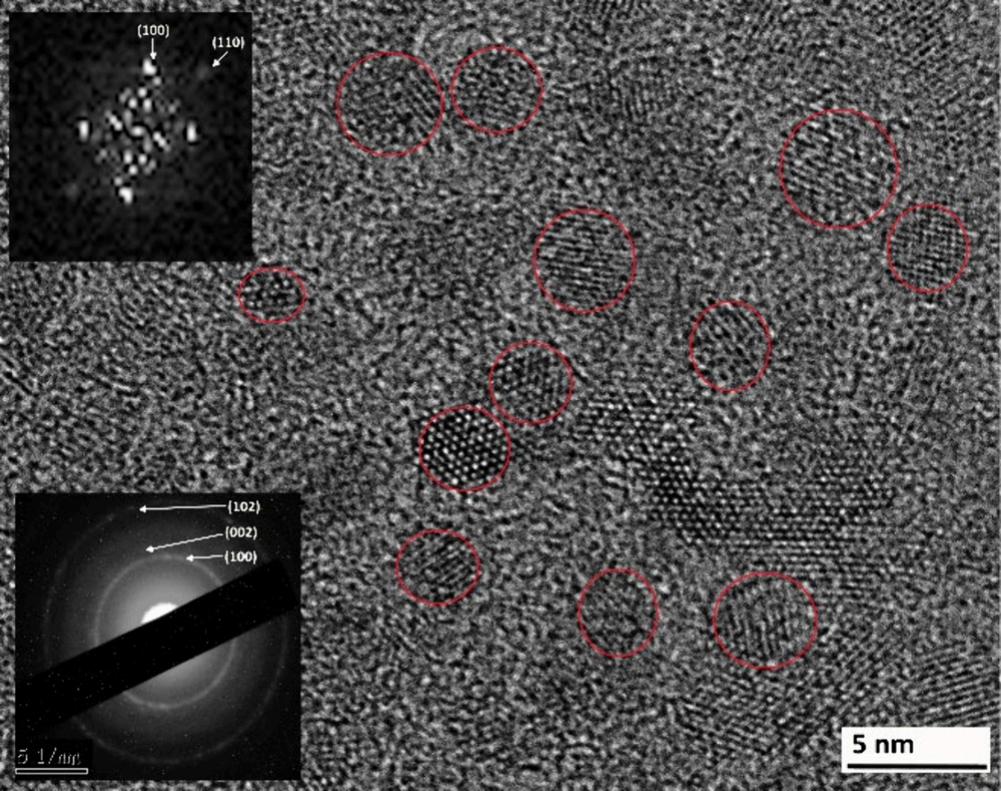
High-resolution transmission electron microscopy (HR-TEM) image of the ZnONPs/PEI N-GQDs nanocomposite, showing crystalline ZnO nanoparticles (∼2–5 nm) embedded in the PEI-functionalized N-GQD matrix. Red circles indicate ZnO lattice fringes, while FFT (upper left) and SAED (lower left) insets confirm the crystalline nature of ZnO. The surrounding low-contrast regions correspond to the semicrystalline or amorphous PEI N-GQDs phase.

As the structure investigated with its all aspects from its bandgap to its composition details, therefore, the potential of a heterostructure of this material on n-type silicon should be analyzed to understand the detailed band structure. The heterostructure optoelectronic device had characterized with Ultraviolet photoelectron spectroscopy (UPS) to determine the band alignment of conductive and semiconductive surfaces by measuring the kinetic energy of photoelectrons ejected by UV light. The result represented an equilibrium energy-band diagram of the Au/ZnONPs/PEI N-GQDs/n-Si heterostructure is shown in Figure 9(a). The band alignment was constructed based on both literature data and the experimentally determined work function of ZnO QDs (Φ = 4.62 eV, obtained from Ultraviolet Photoelectron Spectroscopy (UPS) (Figure 9(b)). Since the work function of Au is higher than that of the ZnO-based composite, a Schottky-type barrier forms at the Au/composite interface, which governs the rectifying behavior of the diode. At the ZnONPs/PEI N-GQDs/n-Si interface, the band arrangement exhibits a staggered (type-II-like) alignment, where electrons transfer from n-Si to the nanocomposite while holes remain confined within the Si layer. These energy-level considerations are consistent with the enhanced photoresponse and detectivity observed experimentally.

**Figure 9:**
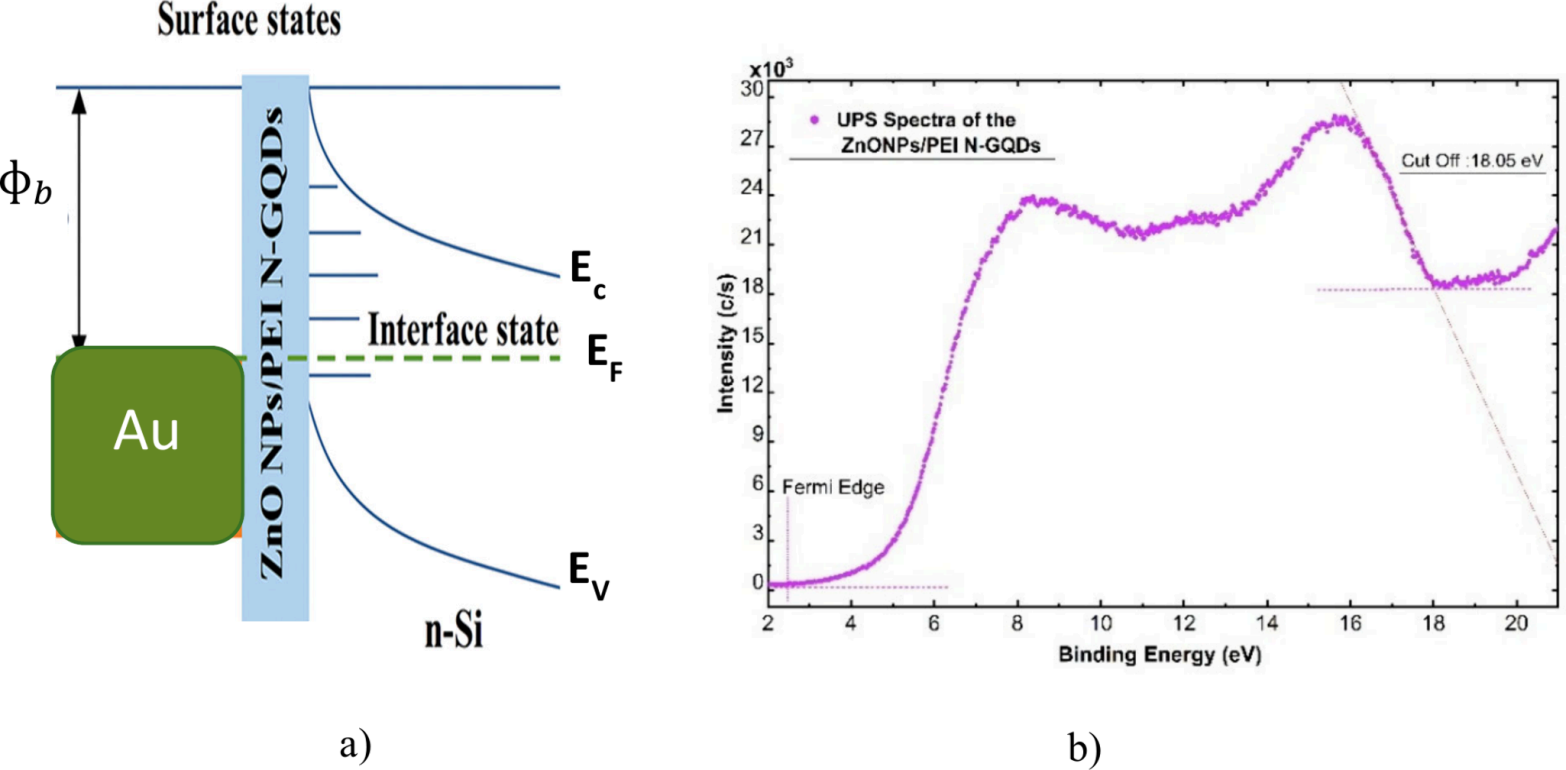
(a) Equilibrium energy-band diagram and (b) UPS spectrum of the ZnONPs/PEI N-GQDs nanocomposite

UPS measurements were conducted determine the work function (Φ) of the ZnO quantum dots (QDs) which illustrated in Figure 9 (b). The Fermi edge (E_Fermi_) and the secondary electron cutoff (*E*_*cutoff*_) were identified by fitting the high and low kinetic energy regions of the UPS spectrum, respectively. The secondary electron cutoff in this investigation was observed at 18.06 eV (BE), while the Fermi edge was identified at 1.46 eV (BE). The whole spectrum was realigned by subtracting the fitted Fermi edge value in order to account for any movement in the Fermi level. This produced a corrected cutoff value of 18.06 eV (BE). The work function (Φ) was then calculated according to *Φ = hν – |E*_*Fermi*_ *– E*_*cutoff*_ *|*. Using this approach, the work function of the ZnO QDs was found to be 3.17 eV, which is consistent with typical literature values for ZnO nanostructures.^14^ These results demonstrate that the role of PEI N-GQDs extends beyond material modification and is primarily associated with interfacial energy alignment and charge-transfer regulation in the heterojunction device. Despite extensive reports on ZnO/GQD and ZnO/polymer hybrid systems, a systematic understanding of how interfacial electronic structure influences charge transport, recombination, and rectification behavior in ZnO-based heterojunction photodiodes remains limited. The present work addresses this gap by employing PEI-functionalized nitrogen-doped graphene quantum dots as an engineered interfacial layer and by correlating UPS-derived energy-level alignment with photoluminescence characteristics and diode electrical performance.

The relatively high ideality factors obtained for the ZnONPs/PEI N-GQDs/n-Si heterojunction indicate the presence of nonideal transport mechanisms beyond pure thermionic emission. Such behavior is widely reported in heterojunction and Schottky-type devices and is commonly associated with interface trap states,^31^ barrier height inhomogeneity,^32^ and recombination-assisted tunneling processes,^33^ particularly in solution-processed and nanostructured heterojunction systems.^34^ In the present device, defect states at the interfacial nanocomposite layer can act as recombination centers and facilitate trap-assisted charge transport, leading to an increase in the apparent ideality factor and deviation from ideal diode behavior. Similar nonideal current–voltage characteristics with elevated ideality factors have been previously reported for ZnO-based and nanostructured heterojunction devices, where interface state density and spatial barrier inhomogeneities dominate the transport mechanism.^31,34^ The elevated ideality factors indicate that charge transport is governed by interface trap-assisted recombination and barrier inhomogeneity effects rather than pure thermionic emission.^31,34^

The present study focuses on the role of interfacial engineering in governing rectification behavior and charge transport in the heterojunction device. A comprehensive evaluation of photodetector performance metrics, including responsivity, detectivity, EQE, and response time, will be addressed in future studies aimed at device optimization.

The staggered energy-level alignment at the ZnONPs/PEI N-GQDs/n-Si interface promotes spatial separation of photogenerated carriers, with electrons preferentially transferred toward the ZnO phase and holes localized within the PEI N-GQDs or the Si substrate1–3. This spatial separation suppresses interfacial recombination and facilitates directional charge transport across the heterojunction.

UPS studies on solution-processed ZnO thin films typically report work function values in the range of ∼ 3.7–4.3 eV, depending on surface states and defect distributions.^26^ The UPS-derived energy-level positions obtained in this study are generally consistent with previously reported values for ZnO and graphene quantum dot–based systems, which vary depending on synthesis conditions and surface states. Reported literature values for ZnO work functions and valence band edges typically fall within an uncertainty range of approximately 0.1 eV, and similar uncertainty ranges have been noted for carbon-based nanostructures. These uncertainties arise from calibration accuracy, surface contamination effects, and fitting procedures inherent to UPS measurements.^35^

### 3.2 Electrical Characterization

Eventually, the potential of the heterostructure device for optoelectronic device had proposed which let the characterization to go deep with electrical characterization. Thermionic emission (TE) theory served as the primary model for extracting fundamental diode parameters such as the ideality factor and barrier height,^18,21^ which provides a fundamental framework for analyzing charge transport in Schottky-type junctions. According to the TE model, the relationship between current and applied voltage in the dark can be described using the following expressions:

1 $$I=I_{0}\left[\exp\left(\frac{q\left(V-IR_{s}\right)}{nkT}\right)-1\right]$$

In this model, *I*_0_, *n*, *V*, *k*, *q*, *IR*_s_, and *T* denote the reverse saturation current at zero bias, the ideality factor, the applied bias voltage, the Boltzmann constant, the elementary charge, the voltage drop across the series resistance *R*_s_, and the absolute temperature in Kelvin, respectively. From the slope of the linear region of the ln­(*I*)–V plot, the ideality factor was determined using

2 $$n=\left(\frac{q}{kT}\right)\left(\frac{dV}{d\ln\left(I\right)}\right)$$

According to the TE or Landauer’s transport theory, the Schottky barrier height (*qΦ*_*B*_) can be extracted from the saturation current *I*_0_ using the following expression:^22^

3 $$\Phi_{B}=kT\ln\left(\frac{AA^{*}T^{2}}{I_{0}}\right)$$

as the saturation current *I*_0_ is given by

4 $$I_{0}=AA^{*}T^{2}\exp\left(-\frac{\Phi_{B}}{kT}\right)$$

where *A* is the effective diode area, A* is the effective Richardson constant (112*A*.*cm* ^–2^ *K* ^–2^ for n type Si). The Φ_B_ and *n* were evaluated using the TE model from the forward-bias ln­(I)–V characteristics according to eqs 2 and ([Disp-formula eq3]) by using the ln­(*I*)–*V* characteristics.

The design and characterization of semiconductor diode structures have become pivotal in the advancement of optoelectronic technologies. Metal–semiconductor (MS) junctions, such as Au/n-type Si, offer simple and well-understood rectifying behavior, serving as a benchmark in electronic device studies. However, recent developments in nanotechnology have enabled the integration of functional nanomaterials like GQDs and metal oxide nanoparticles to form advanced heterostructure diodes with tailored electronic and optical properties.

In this study, two different diode configurations were investigated: a conventional Au/n-type Si MS diode and a heterostructure diode comprising Ag/ZnONPs-embedded PEI N-GQDs/n-type Si. The electrical performance of both devices was analyzed through current–voltage (*I*–*V*) measurements in the dark. The photoresponse measurements of the Au/ZnONPs/PEI N-GQDs/p-Si diode was performed under 1 Sun (AM 1.5G) simulated solar illumination provided by a 300 W xenon (Xe) arc lamp with an intensity of 100 mW/cm^2^, covering the 350–1100 nm spectral range. The light intensity was calibrated using a standard Si reference cell. The main diode parameters, including n, Φ_B_, and RR, were extracted using TE theory and alternative analytical methods to evaluate and compare their rectifying behavior and photoresponse capabilities. The basic diode parameters of the Au/n-type Si diode and ZnONPs/PEI N-GQDs/n-Si heterojunction diode, as extracted using the TE theory, The basic diode parameters extracted using the TE theory are summarized in Table 1.

**Table 1:** Basic Diode Parameters of Au/n-Type Si Diode and ZnONPs/PEI N-GQDs/n-Si Heterojunction Diode

Method	Au/n-type Si diode			ZnONPs/PEI N-GQDs/n type Si diode		
TE	*n*	ϕ_*B*_ (eV)	RR at ±5 V	*n*	ϕ_*B*_ (eV)	RR at ±5 V
Dark	5.06	0.70	2.17 × 10^4^	9.79	0.72	3.25 × 10^3^
Illuminated-100mW/cm^2^	3.16	0.76	3.04 × 10^2^	3.17	0.76	6.64 × 10^1^

At 300 K, the Au/n-type Si diode exhibited a higher RR (*I*_*forward*_ */|I*_*reverse*_ *|*) in the dark (2.17 × 10^4^ at ± 5 V) and a lower ideality factor (n = 5.06), whereas the heterojunction diode showed enhanced photoresponse under illumination, with its ideality factor improving to 3.17 and the barrier height reaching 0.76 eV. Despite a reduction in RR under illumination for both devices, the heterostructure demonstrated strong light sensitivity, highlighting its potential for optoelectronic and photodetection applications. The relatively high ideality factor (*n* > 2) arises from interface states, barrier inhomogeneities, and recombination processes within the ZnONPs/PEI N-GQDs/p-Si heterostructure, which deviate from ideal thermionic emission behavior.

The semilogarithmic current–voltage (ln *I*–*V*) characteristics of the Au/n-type Si and ZnONPs/PEI N-GQDs/n-Si heterojunction diodes, measured at 300 K under dark and 100 mW/cm^2^ illumination conditions, are presented in Figure 10. Figure 10(a) displays the ln *I*–*V* characteristics of the Au/n-type Si Schottky diode. The ln *I*–*V* characteristics of the Au/n-type Si Schottky diode measured at room temperature under dark and illuminated conditions show clear rectifying behavior. Under illumination, the forward current increases significantly compared to the dark condition due to the generation of additional charge carriers. The increase in reverse current under illumination indicates a strong photoresponse, confirming the potential of the device for optoelectronic applications. The RR of the Au/n-type Si diode significantly decreases under illumination, dropping from 2.17 × 10^4^ in dark conditions to 3.04 × 10^2^ at ±5 V under 100 mW/cm^2^ light exposure. The significant drop in RR under illumination is primarily due to the increase in reverse current. Light generates excess electron–hole pairs, which enhance the reverse leakage current. As a result, the *I*_*reverse*_ increases while *I*_*forward*_ remains relatively stable, leading to a lower RR.

**Figure 10:**
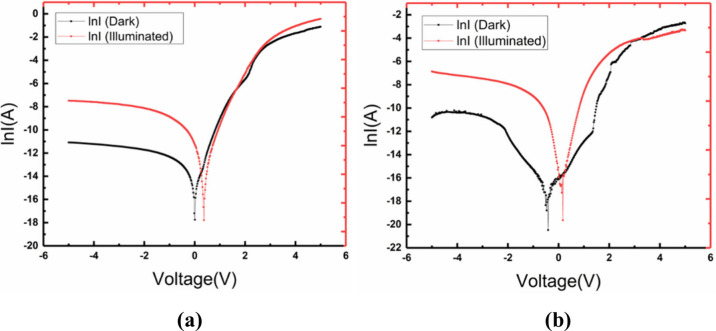
(a) ln *I*–*V* characteristics of Au/n-type Si. (b) ZnONPs/PEI N-GQDs/n-type Si heterojunction diodes under dark and 100 mW/cm^2^ illumination conditions.

The ln *I*–*V* characteristics of the ZnONPs/PEI N-GQDs/n-type Si heterojunction diode, measured under dark and 100 mW/cm^2^ illumination, are shown in Figure 10(b). The ln *I*–*V* characteristics of the Au/ZnONPs/PEI N-GQDs/n-type Si heterojunction diode exhibit pronounced rectification behavior and a significantly enhanced photoresponse compared to the Au/n-Si reference device. Under illumination, a considerable increase in both forward and reverse currents is observed, attributed to photogenerated carriers facilitated by the ZnONPs/PEI N-GQDs interlayer. The strong suppression of dark reverse current, along with the enhanced photocurrent under light exposure, confirms the heterostructure’s high photosensitivity and makes it a promising candidate for photodetector and photovoltaic applications. For the heterojunction diode, the RR decreased from 3.25 × 10^3^ in the dark to 6.64 × 10^1^ at ±5 V under 100 mW/cm^2^ illumination, indicating a slight compromise in rectification performance. Similarly, both diodes exhibited a noticeable reduction in RR under illumination due to a significant increase in reverse current caused by photogenerated carriers. Under illumination, the filling of interface trap states reduces the potential barrier asymmetry between forward and reverse bias, leading to an increased reverse current and a reduced rectification ratio. Various studies have reported a broad range of diode parameters for ZnO-based structures, reflecting differences in material processing, contact metals, and measurement conditions. The Schottky barrier height values reported in four studies span from as low as 0.3 eV to as high as 1.2 eV. The lowest barrier height, 0.3 eV, was observed by^23^ using a nickel contact under high drain-source voltage, whereas the highest value of 1.2 eV was recorded by^24^ at 400 K. The ideality factor, available in three studies, exhibited significant variation between 1.15 and 9.8. The lowest ideality factor (1.15) was obtained under low electric field conditions by,^23^ while the highest (9.8) was observed at a low temperature of 100 K in the study by.^24^ The RR also varied widely, with values ranging from 2 for unembedded ZnO to above 10^6^, as reported by Sinha et al.^25,26^ Additional findings include rectification ratios between 1.13 × 10^2^ and 7.564 × 10^3^, and up to 10^4^ for embedded ZnO structures.^27,36^ Studies report that interface features—such as oxygen vacancies, electron traps, and barrier inhomogeneities—govern electron transport primarily through tunneling, thermionic emission, and recombination mechanisms^36,37^ Several studies reported strong rectification ratios and significant ultraviolet (UV) photoresponse in ZnO nanoparticle-based diodes.^36^ For example, rectification ratios ranged from approximately 6 to 2000, and some devices showed a transition from rectifying to ohmic behavior under UV illumination −366 nm light.^38^

The Au/ZnONPs/PEI N-GQDs/n-Si heterojunction exhibits an ideality factor of approximately 9.79 in the dark, which deviates significantly from ideal thermionic emission behavior. This nonideal behavior is attributed to the presence of interface defect states, trap-assisted recombination, and barrier inhomogeneities introduced by the ZnONPs/PEI N-GQDs interfacial layer. Under illumination, the ideality factor decreases to 3.17, suggesting that photogenerated carriers fill trap states and improve the effective potential uniformity across the junction. The improvement in carrier transport under illumination aligns with the enhanced EQE response, which indicates more efficient charge collection due to interfacial passivation and strong photoinduced carrier transfer. Although impedance spectroscopy was not conducted in this work, the correlation between ideality factor and EQE response supports the proposed mechanism involving interfacial defect-state modulation.^27,39^

Compared to previously reported ZnO-based Schottky diodes, the ZnONPs/PEI N-GQDs/n-type Si diode developed in this study exhibits several comparable and in some cases superior characteristics. The barrier height of the fabricated diode was measured as 0.72 eV in the dark and 0.76 eV under illumination, which falls well within the literature-reported range of 0.3–1.2 eV. These values are notably close to the upper end, indicating a stable and effective metal–semiconductor interface. The ideality factor, although relatively high in the dark (9.79), significantly decreased to 3.17 when illuminated, suggesting improved carrier transport under light exposure—an improvement that is not commonly highlighted in ZnO-based devices, where reported values range from 1.15 to 9.8. The rectification ratio (RR) of the device was 3.25 × 10^3^ in the dark, which aligns with or exceeds those of many unembedded ZnO diodes and is comparable to embedded structures^40^ that reach up to 10^4^. Under illumination, the RR decreased to 6.64 × 10^1^, which is lower than the extreme values higher than ten to power of 6 but remains within the effective operational range reported in the literature.^41^ Overall, the presented diode demonstrates barrier height stability, moderate-to-high rectifying behavior, and a strong photoresponse, positioning it as a competitive candidate among ZnO-based Schottky structures.^42^

The photoresponse characteristics of the fabricated diode were quantitatively analyzed to determine its sensitivity to incident light in terms of responsivity (R) and specific detectivity (*D* ^*^).

5 $$R=\frac{I_{light}-I_{dark}}{P_{in}.A_{d}}$$

where *I*_*ph*_ is the photocurrent, *P*_*in*_ is the incident optical power density (100 mW·cm^–2^), and *A*_*d*_ is the illuminated device area (cm^2^). It represents the detector’s ability to convert incident optical power into electrical current, with larger responsivity values corresponding to more efficient photon-to-electron conversion. D* offers a comprehensive assessment of the device’s capability to sense weak optical signals by incorporating noise-related effects. D* was determined using standard equations commonly employed in recent reports.^42^

6 $$D^{*}=\frac{\sqrt{A_{d}\times \Delta f}}{NEP}=R\times \frac{\sqrt{A_{d}\times \Delta f}}{I_{noise}}$$

where *A*_*d*_ is the photosensitive area of the photodetector, and *Δf* is the bandwidth (which is equal to 1 Hz). In some cases, when the noise is mainly shot noise, (2*qI*_dark_)^1/2^ is usually used to calculate the theoretical noise current (*I*_noise_) of the photodetector, where *I*_dark_ is the dark current of the detector. *D* ^*^ often expressed in with Jones unit that stands for cm·Hz^1/2^/W in SI unit.

After evaluating the responsivity and specific detectivity, the external quantum efficiency (EQE) was calculated to further quantify the photoresponse capability of the device and to express the number of charge carriers generated per incident photon.

It is defined as

7 $$EQE\;\left(\%\right)=\frac{R\times 1240}{\lambda }$$

where *R* is the responsivity (A.W^1–^) and λ is the wavelength of the incident light (nm).

The constant 1240 corresponds to *hc*/*q*, derived from Planck’s constant (*h*) and the speed of light (*c*). This relation directly connects the electrical and optical responses of the photodiode, serving as a measure of its photon-to-electron conversion efficiency. In this study, the ZnO-embedded GQDs/n-Si heterojunction photodiode exhibited a peak responsivity of 1.16 A/W under visible illumination at 550 nm.

The calculated *R*, D* and EQE values are shown in Figure 11(a–c), respectively. The device exhibited a maximum responsivity of approximately 1.16 A.W^1–^ and a detectivity of about 5 × 10^10^ Jones at −5 V, indicating excellent photoresponse characteristics. Figure 1(c) shows the variation of the EQE of the ZnO-embedded GQDs/n-Si heterojunction photodiode within the reverse-bias region (−5 to 0 V). The EQE decreases sharply from approximately 262% at – 5 V to nearly zero at 0 V. This strong bias dependence indicates that the efficient carrier separation and photoconductive gain observed at higher reverse bias vanish as the internal electric field collapses near zero bias. This behavior is attributed to the reduction in the internal electric field strength and the corresponding decrease in carrier-collection efficiency. The smooth and monotonic trend in this region confirms stable diode operation with low noise and reproducible photoresponse.

**Figure 11:**
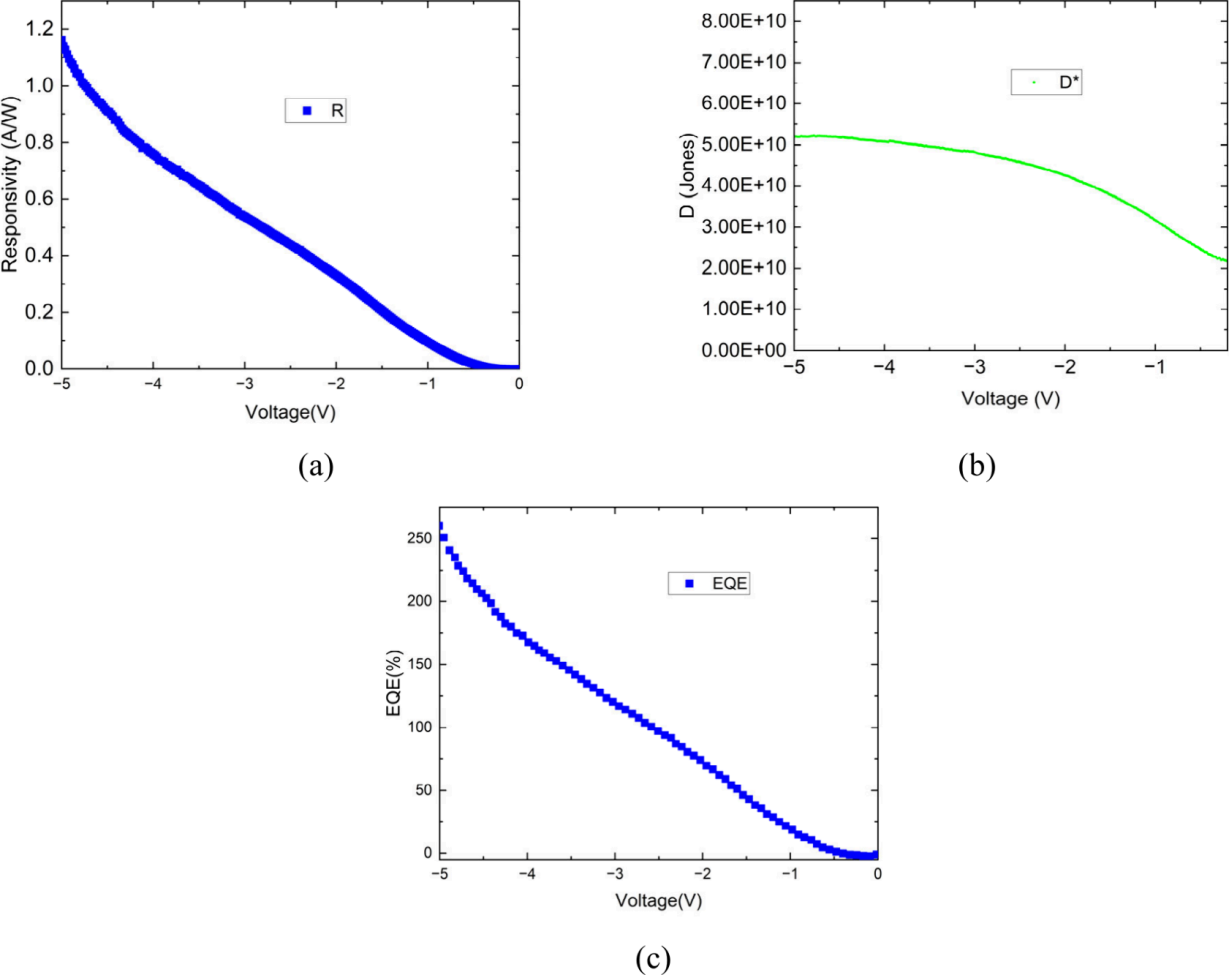
(a) Responsivity, (b) detectivity, and (c) EQE (external quantum efficiency) of the Ag/ZnONPs PEI N-GQDs/n-Si heterojunction.

Because absolute photodiode performance metrics reported in the literature are obtained under substantially different device architectures, bias conditions, and illumination protocols, a relative comparison based on order-of-magnitude trends is adopted. Compared to ZnO nanorod/GQD Schottky photodiodes, which exhibit responsivities on the order of several tens of A.W^1–^ due to nanorod-induced photoconductive gain, the responsivity of the present ZnONPs/PEI N-GQDs/n-Si device is lower by approximately one to 2 orders of magnitude; however, it is achieved within a planar, solution-processed heterojunction architecture without relying on nanostructure-enabled carrier multiplication effects.^25^

In comparison with ZnO:GQD/Poly-TPD heterojunction devices, which typically show detectivity values on the order of 10^11^ Jones, the detectivity of the present device lies within the same order of magnitude, despite the absence of an additional organic charge-transport layer.^15^ When benchmarked against GQD-decorated ZnO/GaN heterojunction photodetectors, where wide-bandgap epitaxial substrates enable detectivities approaching the 10^12^ Jones regime, the responsivity of the present device is approximately order of magnitude lower; nevertheless, the performance remains competitive considering the fully solution-processed interface and the use of a conventional n-Si substrate.

In contrast, relative to ZnO superstructure/GQD photodetectors that primarily emphasize switching behavior and on/off ratio rather than absolute responsivity or detectivity, the present device demonstrates a higher effective photodiode response, highlighting the advantage of interfacial energy-level engineering over purely morphological optimization strategies.

## 4 Conclusions

This work demonstrates a green, solution-based synthesis of a ZnONPs/PEI N-GQDs nanocomposite and its successful integration as an interfacial layer in an n-Si heterojunction photodiode. UV–Vis spectroscopy and Tauc-plot analysis reveal a direct band gap of ∼3.0 eV (≈409 nm), red-shifted relative to pristine ZnO, while PL emission centered at ∼518 nm (∼2.48 eV) indicates defect- and interface-mediated radiative recombination between ZnONPs and PEI-functionalized N-GQDs. FTIR and XPS jointly confirm the hybrid structure, showing PEI-linked N- and O-containing surface groups together with Zn–O lattice modes and Zn^2+^, pyrrolic-N, and graphitic-N states. HR-TEM further reveals 3–5 nm crystalline ZnO nanoparticles embedded within a semicrystalline N-GQD matrix, evidencing well-defined nanoscale heterointerfaces. UPS-derived work-function data (Φ ≈ 3.17 eV) support a Schottky contact at the Au/composite interface and a type-II-like, The staggered (type-II-like) energy-level alignment at the ZnONPs/PEI N-GQDs/n-Si interface promotes spatial separation of photogenerated carriers, with electrons preferentially transferred toward the ZnO phase and holes localized within the PEI N-GQDs or the Si substrate This spatial separation suppresses interfacial recombination and facilitates directional charge transport across the heterojunction. Band alignment at the ZnONPs/PEI N-GQDs/n-Si junction, favorable for carrier separation.

Electrically, the Au/ZnONPs/PEI N-GQDs/n-Si diode exhibits clear rectification and pronounced photosensitivity. Compared to the Au/n-Si reference (RR = 2.17 × 10^4^, *n* = 5.06), the heterojunction shows a moderate rectification ratio (3.25 × 10^3^ in the dark; 6.64 × 10^1^ under 100 mW cm^–2^ illumination) but an improved illuminated ideality factor (*n* = 3.17) and a barrier height of 0.76 eV. The device achieves a peak responsivity of ∼1.16 A W^1–^, a detectivity of ∼5 × 10^10^ Jones, and an EQE up to ∼262% at −5 V, underscoring efficient photocarrier generation, separation, and collection. Overall, these results highlight ZnONPs/PEI N-GQDs nanocomposites as promising, solution-processable interlayers for silicon-based optoelectronic and photodetector technologies.

## Data Availability

Data used are available throughout the manuscript text.
